# Predicting enzyme-compound associations for enzyme-catalysed reactions

**DOI:** 10.1186/s13321-026-01190-w

**Published:** 2026-04-22

**Authors:** Liam Brydon-Brown, Gillian Dobbie, Katerina Taškova, Jörg Simon Wicker

**Affiliations:** 1 School of Computer Science, University of Auckland, Auckland, New Zealand; 2 enviPath Limited, University of Auckland, New Zealand

**Keywords:** Cheminformatics, Biodegradation, Hierarchical learning, Enzyme prediction, Product prediction

## Abstract

Enzyme-catalysed reactions are common in many areas, including pharmaceutical metabolism and agricultural chemical biodegradation. Analysing and predicting how these reactions occur is increasingly important for identifying toxic by-products and achieving regulatory approval. Incorporating enzyme information into these predictions has been shown to improve prediction capabilities. However, existing methods require knowledge of the enzyme to perform prediction, and in many situations, especially biodegradation, the complexities of the reaction environment mean the exact enzymes are not known. In this paper, we alleviate this issue by proposing a framework to train and evaluate a hierarchical multi-label classifier to predict the association between enzyme commission numbers and chemical compounds. Our method achieves a hierarchical F1-score of up to 93.2%, outperforming existing methodologies. Additionally, we examine how including true and predicted enzyme information impacts product prediction performance compared to not using enzyme information. In our case study utilising biodegradation reaction data, we find that including enzyme commission numbers improve product prediction performance by approximately two percentage points.

**Scientific contribution**

We contribute a novel method for predicting enzyme-compound associations using a hierarchical multi-label classifier framework. Our method is self tuning to find the best hyperparameters for a given dataset and achieves higher F1 scores than existing methods. We also contribute an investigation into including enzyme information into product prediction algorithms, showing that including this information can improve product prediction performance.

**Graphical abstract:**

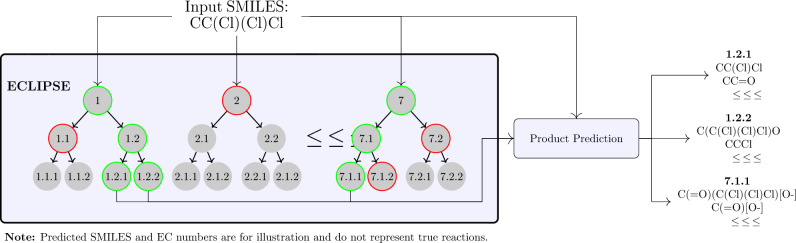

**Supplementary Information:**

The online version contains supplementary material available at 10.1186/s13321-026-01190-w.

## 1 Introduction

Predicting the products of chemical reactions is important for analysing how chemicals behave in the environment. For drug metabolism, performing these predictions allows chemists to potentially identify toxic metabolites before clinical testing [1]. Most recently, regulations have begun requiring biodegradation modelling to be submitted to obtain regulatory approval for any novel chemical [2]. Traditional lab experiments to analyse chemical behaviour are expensive and time-consuming. Subsequently, there has been a large research focus on developing machine learning methods to perform product prediction.

When dealing with chemicals in natural environments, it is extremely common for the reactions to be enzyme-catalysed, where an enzyme interacts with a compound to cause a change. The class of enzyme, defined by the Enzyme Commission (EC) number [3], plays a significant role in determining what reaction will occur. Existing product prediction methods largely ignore the enzyme class instead relying solely on the reactant chemical to perform prediction, commonly using sequence representations of compounds like the Simplified Molecular Input Line Entry System (SMILES) [4]. While some work has been done to include enzymes in product prediction inputs, it is limited to joining the EC number with the reactant SMILES and assumes the enzyme class is known [5, 6]. For complex environments such as those in biodegradation, the enzyme classes are not always known, thus limiting the applicability of such methods. Recent works tackles this by taking a whole reaction containing the SMILES of both reactants and products and predicting the enzyme class [7, 8]. However, these methods create a circular dependency where predicting the product requires knowing the enzyme and predicting the enzyme requires knowing the product. This paper breaks the circular dependency by predicting the EC number(s) associated with a given compound, rather than the EC number of the enzyme that caused a reaction. The EC number is represented by up to four numbers separated by dots, for example, 1.2.2.5. This is a hierarchical system where each position in the number is a subclass of the previous position. Subsequently, we propose the Enzyme Class Learning and Interaction Prediction SystEm (ECLIPSE), a framework for training and evaluating a hierarchical multi-label EC number classifier. ECLIPSE’s primary function is to take in a compound and predict one or more EC numbers.

After developing and evaluating ECLIPSE, we test how including EC numbers influences product prediction performance. We do this with the Transformer architecture used by many existing works [9–14] and follow previous research in this area by concatenating the EC number to the reactant SMILES [7, 8]. In our product prediction analysis, we compare the performance of using the known EC numbers against no EC numbers and our predicted EC numbers. Comparing to our predicted EC numbers allows us to assess how the propagation of errors from ECLIPSE to the product prediction affects overall product prediction performance.

We summarise our contributions as follows:

- A hierarchical multi-label classification method for training and predicting enzyme-compound associations.
- An evaluation of how enzyme information impacts product prediction performance.
- A case study of enzyme and product prediction on small biodegradation datasets.

## 2 Related work

In this section, we summarise previous works tackling similar problems. In Section 2.1, we look at reaction-level enzyme prediction methods, followed by existing work in hierarchical classification in Section 2.2 and how we can apply it to our problem. Lastly in Section 2.3, we summarise how enzyme information is incorporated to improve product prediction performance.

### 2.1 EC number prediction

Previous EC number prediction research primarily focuses on development of methods that predict EC numbers from a reaction. This differs to our goal of predicting EC numbers solely from a compound (without the product of the reaction). These methods often use reaction fingerprints as features, since we are only inputting a compound we are unable to use these methods directly.

Qian et al. [7] looked at predicting the EC number of the enzyme that caused a given reaction. They use a BERT [15] style transformer model that takes the reactant and product SMILES as inputs and performs classification to get the EC number. Their method achieves an F1-score of 91.3% on their task.

The Theia method published by Probst [16] provides a multilayer perceptron method for predicting the EC number of a reaction. Theia uses a differential reaction fingerprint to encode the reaction for model input. They combine their predictions with DeepLIFT [17] to show which parts of the input molecule contributed to the predicted EC class, providing a high level of explainability for their predictions.

Another method by Zeng et al. [8] used a contrastive learning approach combined with the reaction fingerprint RXNFP [18] to predict the EC number of a reaction. Contrastive learning aims to put data of the same class close together and different classes far apart in an embedding space. Prediction is then performed by finding the nearest known point to the predicted point in the embedding space and assigning the known point’s class to the prediction. Using this method, they achieve a weighted F1-score of 90%.

Compound-protein interaction has also been explored in recent work. Wang et al. [19] proposed an attention-based Transformer method that utilises transfer learning to address multiple enzyme interaction tasks. They utilise transfer learning to overcome data sparsity in their dataset. Methods for overcoming data sparsity are useful in enzyme-compound datasets where data is often limited.

### 2.2 Hierarchical classification

The hierarchical nature of EC numbers suggests that hierarchical classification methods are appropriate. Additionally, as multiple EC numbers can be associated with a compound, a multi-label version would be most suitable. The main benefit of utilising a multi-label hierarchical classifier is that errors can often be confined to a single lineage of the hierarchy. In the context of EC prediction, a mistake at the third EC level could still yield a biologically meaningful EC prediction, since the first two levels are correct. Such approaches have been applied in various domains, including the prediction of protein and gene function [20, 21].

There are three main ways hierarchical classifiers are built, Local Classifier per Level (LCL), Local Classifier per Parent Node (LCPN) and Local Classifier per Node (LCN) [22]. LCL trains one multi-class model per level to predict the class at that level, whereas LCPN trains a multi-class model per parent node to predict the child class. In contrast to LCL and LCPN, LCN trains a binary classifier on every node in the hierarchy. Subsequently, LCN is more naturally suited to a multi-label hierarchy as multiple classifiers at a level could return positive predictions, compared to the multi-class of LCL and LCPN, where the goal is generally to predict one class.

### 2.3 Product prediction with enzyme information

Recent works have looked at incorporating enzyme information into reaction prediction for enzyme-catalysed reactions. These papers concatenate up to the third digit of the EC number to the end of the reactant, then use the commonly used transformer-based product prediction method to learn from this SMILES-EC input [5, 6]. They found that including the EC numbers improves reaction prediction performance.

## 3 Method

In this section, we first detail the datasets we use (Section 3.1) and how we preprocess them (Section 3.2). Following that, we give our method for performing EC number prediction, ECLIPSE, in Section 3.3 and how we incorporate the predicted enzymes into product prediction in Section 3.4. Lastly, Section 3.5  explains the metrics we use for evaluating ECLIPSE and the product prediction method.

### 3.1 Datasets

**Fig. 1:**
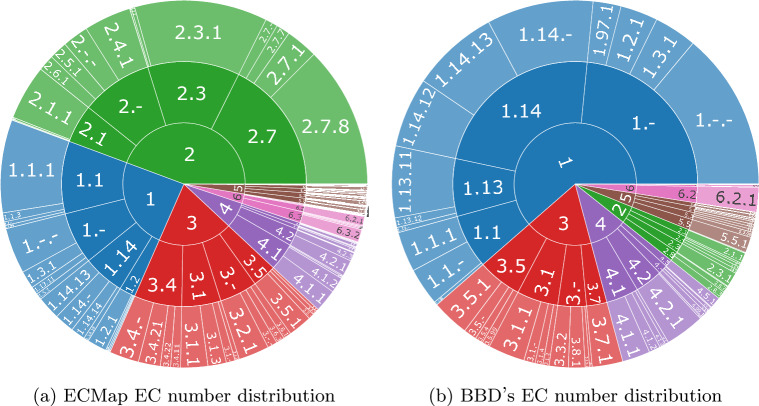
Distribution of EC numbers in ECMap (**a**) and enviPath’s BBD (**b**). We show the top three most common classes at the second and third level and group the rest into the dash. The colour segments each of the seven level one EC numbers

We use two main datasets in developing our method. These include ECMap [5, 6], and enviPath [23, 24] datasets. A summary of the distribution of these datasets is given in Fig. 1. We give the multi-label statistics of cardinality, density and distinct in Table 1. Cardinality is the average number of labels per instance. Density is cardinality divided by the number of labels. Distinct is the number of unique multi-label sets.

#### ECMap

ECMap contains $\sim 100,000$ enzyme catalysed reactions. We have created this dataset by combining the ECREACT dataset [6] and the EnzymeMap dataset [5]. Those datasets were originally extracted from sources such as BRENDA [25], KEGG [26], MetaNetX [27] and more. We removed any duplicate reactions by comparing reaction SMILES standardised with Rdkit [28]. This dataset gives the EC number alongside the reaction SMILES. Figure 1a shows that the distribution of the first EC number consists mainly of EC classes one, two and three, with significantly smaller amounts of four through seven. Table 1 shows that ECMap is very close to a single-label dataset with low cardinality and density values, and only 4.1% multi-label samples. This is the primary dataset we use to train and evaluate our methods.

#### enviPath

EnviPath contains $\sim 4,400$ biodegradation reactions across three packages, Soil [29], Biocatalysis/Biodegradation Database (BBD) [30] and Sludge [31]. However, only the BBD package contains significant EC information, with about 1,300 reactions containing ECs, whereas Soil and Sludge contain approximately 10 reactions with EC information. Figure 1b shows a very different distribution compared to ECMap, with EC classes one and three making up the vast majority of the dataset. Looking at the statistics in Table 1, BBD is more multi-label than ECMap with 10.4% multi-label samples, but it is still largely a single-label dataset. As enviPath is substantially smaller than ECMap, we use it as a case study to see how smaller datasets change the performance of our EC number prediction method and the performance of incorporating the EC number into product prediction.

**Table 1:** Multi-label dataset metrics for ECMap and BBD

Metric	ECMap	BBD
Num-Samples	82,614	1022
Multi-label Samples	3408 (3.69%)	106 (10.37%)
Unique ECs	155	16
Cardinality	1.053	1.117
Density	0.007	0.070
Distinct	1153	65

### 3.2 Preprocessing

For preprocessing, we use only the first three digits of the EC number. This is in line with the methods used to predict ECs for a reaction [8, 16]. We then ensure a minimum of 40 samples are available for each EC number; any fewer and our train/val/test split, described in Section 3.6, would not have adequate instances. Where there is not enough data available, we replace the last digit of the EC number with a dash, effectively grouping them (i.e 3.2.1 and 3.2.2 could be grouped into 3.2.-). This is done so we can retain as much data as possible. However, in the case of BBD, there is insufficient EC six and EC seven data, and therefore, a small number of instances are removed.

### 3.3 EC number prediction

Our EC number predictor, ECLIPSE, aims to take a compound SMILES as the input and classify the EC number(s) most likely associated to the compound. Our method is adaptive by utilising a tuning process to pick the best hyperparameters for the given dataset. We propose two versions of ECLIPSE, the Hierarchical ECLIPSE (H-ECLIPSE), described in Section 3.3.2 and the Flat ECLIPSE (F-ECLIPSE), described in Section 3.3.3.

#### 3.3.1 SMILES processing

For the F-ECLIPSE and H-ECLIPSE input, we convert molecule SMILES into molecular fingerprints that encode the structures of the molecule in a fixed-length vector. We opt to use the Morgan Fingerprint [32] implemented in rdkit [28] with the default values for radius (2) and fingerprint size (2048). We chose this as it is a commonly used fingerprint that provides a rich representation of the molecule’s features. Additionally, research has shown that most molecule fingerprint methods are equivalent and the exact fingerprint is unlikely to change the method’s performance [33]

#### 3.3.2 Hierarchical classifier

**Fig. 2:**
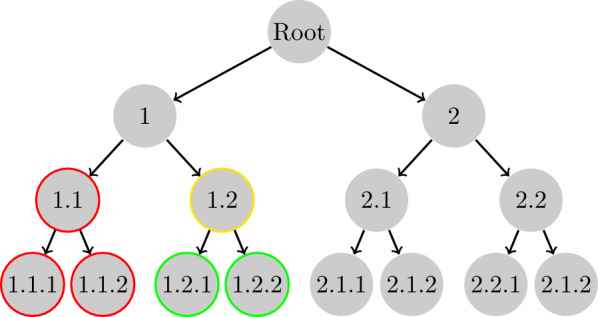
The siblings binary-policy with the yellow outline node as the node being trained. Green nodes are positive examples, red nodes are negative examples and non-outlined nodes are not used for training

To develop our H-ECLIPSE, we use a hierarchical multi-label classification algorithm with LCN (as described in Section 2.2). We use the decision tree ensemble method XGBoost [34] for each node’s classifier. As we use LCN, we need a binary training policy for each node. We use the *sibilings* policy for training the classifier on each node. This is where a subset of the whole dataset is used to train a given node. In Fig. 2, the yellow outlined node is the node being trained. Only data from the classes associated with the given node’s descendants, siblings, and siblings’ descendants are used during training, these are all the outlined nodes. From this subset, the positive examples are those belonging to the given node and its descendants, the green outlined nodes in Fig. 2. The negative examples are those of the siblings and their descendants, the red outlined nodes in Fig. 2. We chose this policy as it helps balance the subset of data used for training each node. Other policies use data from other branches in the hierarchy (the non-outlined in Fig. 2), thus increasing the size of the negative class used to train each node. This disproportionately sized negative class would likely harm each classifier’s performance.

When used for inference, all models at the first level are given the molecular fingerprint of the input SMILES. We then find the highest probability and subtract a *tolerance* value. All classes predicted with a probability greater than that are taken as the predicted classes at level one. For each predicted class at level one, the models associated with the children of the predicted level one classes are used with the same process. After performing this at each level in the hierarchy, the final prediction(s) are given. This method guarantees that at least one prediction will be made and allows us to tune the tolerance value (described in Section 3.3.4).

#### 3.3.3 Flat classifier

The F-ECLIPSE uses an Ensemble of Classifier Chains (ECC) with XGBoost as the individual classifier. Instead of treating the prediction as a hierarchy, the F-ECLIPSE ‘flattens’ the EC hierarchy into a standard multi-label classification problem. The F-ECLIPSE is trained with the same classes as the hierarchical classifier. This version of our method primarily serves as a simple baseline to compare to the H-ECLIPSE.

For inference, we perform one prediction with the F-ECLIPSE, giving probabilities for each class. We then use the tolerance to find the positive instances, classes with probabilities above $\max (probs) - \text {tolerance}$, the same process occurring at each H-ECLIPSE level.

#### 3.3.4 Hyperparameter tuning process

The H-ECLIPSE and F-ECLIPSE utilise a tuning process to determine the best values for their respective hyperparameters. Both of them tune the number of estimators in XGBoost, the maximum depth of those estimators and the tolerance value. Additionally, F-ECLIPSE also tunes the number of chains in its ensemble. The value choices for these parameters are given in Table 2. We perform this tuning by creating a stratified validation split from the training data to estimate the performance of each configuration on unseen data. The tuning process utilises a simple grid search over all possible combinations of parameters. After each configuration is trained, we calculate performance metrics for each configuration using the validation data. The model whose parameters achieved the highest hierarchical F1-score (described in Section 3.5.1) is selected as the best model.

**Table 2:** ECLIPSE tuning parameters and their options

Method	Parameter	Choices
F-ECLIPSE & H-ECLIPSE	Num Estimators	{20, 50, 100, 200, 500}
	Max Depth	{3, 6, 9, 12, 15}
	Tolerance	{0, 0.01, 0.025, 0.1, 0.4}
F-ECLIPSE	Num Chains	{5, 10, 20, 40}

### 3.4 Product prediction

We pretrain an encoder-decoder transformer model [9] on the United States Patent and Trademark Office dataset for our product prediction method [35]. The United States Patent and Trademark Office dataset is a commonly used generic reaction dataset that contains approximately one million reactions. Following existing research, we treat the product prediction problem as a sequence-to-sequence translation task where the product SMILES must be predicted given the reactant SMILES [10, 36]. We preprocess all the reaction SMILES by first canonicalising them with rdkit [28]. The canonicalising ensures all SMILES are formatted the same. We also keep stereochemistry present in all our datasets. We separate all the reactions into reactants and products by splitting them on the “$>>$" token. We then tokenise each side of the reaction using the regular expression below. If either side of the reaction is longer than 380 tokens, we discard the reaction.

$$\begin{aligned} [\vert ]|[A-Z][a-z]?|[A-Z]|[a-z]|:[0-9]{3}|:[0-9]{2}|:[0-9]|[0-9]|(|)|.|=|\#|-|+|\backslash |/|:|\sim |@@|@|?|\rangle \rangle |\rangle |*|\$|\% \end{aligned}$$

The parameters of our pretrained model are taken from our previous work [12] and are given in Table 3. We then refine the pretrained model with the ECMap data, concatenating the actual EC class number to the reactant.

**Table 3:** Our pretrained product prediction transformer parameters

Parameter	Value
Encoder Layers	4
Decoder Layers	4
Heads	8
Embedding Dimension	256
Feedforward Dimension	2048
Dropout	0.1
Max Sequence Length	380

We only use the EC numbers predicted by ECLIPSE during inference. For training, we use the true EC numbers to ensure the product prediction model gets the most accurate information.

### 3.5 Evaluation metrics

We use different evaluation metrics for ECLIPSE and for the product prediction evaluation. These are detailed in Sects. 4.5.1 and 4.5.2.

#### 3.5.1 ECLIPSE metrics

Since we treat the EC number prediction as a hierarchical multi-label classification problem, we use precision, recall and F1-scores specifically designed for hierarchical multi-label tasks [37]. The difference between these and their regular counterparts is the ability to be partially correct. If a prediction gets EC levels one and two correct but not three, it still contributes two-thirds to the relevant metric. The contribution is calculated by converting each hierarchical class to a set containing the label at each level and then checking the intersection size between the true and predicted sets. Equations 1, 2, and 3 formalise these calculations where *C* and $C'$ are the true and predicted sets, respectively.

1 $$\begin{aligned} \text {Precision} = \frac{|C' \cap C|}{|C'|} \end{aligned}$$

2 $$\begin{aligned} \text {Recall} = \frac{|C' \cap C|}{|C|} \end{aligned}$$

3 $$\begin{aligned} \text {F1} = \frac{2 \times \text {Precision} \times \text {Recall}}{\text {Precision} + \text {Recall}} \end{aligned}$$

In our multi-label scenario, the true or predicted set could contain numerous classes at each level. It is possible to weight the errors made at each level of the hierarchy differently, however, we opt to keep them equal.

In addition to the hierarchical measures, we also calculate precision, recall and F1-scores at each EC level. These per-level metrics differ from the hierarchical as they require the first ‘X’ EC digits to match and do not give partial points for correct parent classes. For example, we take only the first two digits of the true and predicted EC class when evaluating at EC level two. These are then compared against each other, checking for equality.

#### 3.5.2 Product prediction metrics

We use both Top-K and precision-recall (PR) curves to evaluate the product prediction model. Top-K gives an overall accuracy of the K most likely sequences that the model gives, and is commonly used in the existing research. However, we showed in our previous work that Top-K has significant flaws [12]. Most importantly, it ignores the drop in precision associated with higher K values caused by the increased number of predictions. Therefore, it is akin to recall, not accuracy. Secondly, Top-K also ignores the specific probabilities given to sequences. By simply ranking them, it is assumed that all predictions at a given K have the same probability, this is likely not the case. Therefore, we also use PR curves to evaluate the predictions described in our previous work [12]. When comparing to PR curves, we say one curve is better than another if it is more towards the top right corner of the plot (precision and recall of one), and also compare the peak precision and recall values of each curve.

### 3.6 Experiment design

For all experiments, we stratify our train/test splits using a multi-label stratification based on the level three enzyme classes that are associated with each reactant compound [38]. We use a 10-fold stratified cross-validation for all experiments and report the mean and standard deviations of all our metrics. Following our methodology in Section 3.3.4, the tuning process occurs ten times, potentially giving different configurations across the different cross-validation folds. When we do product prediction, we use the same 10-folds as the ECLIPSEs are trained on to ensure no advantage is given to the ECLIPSEs by providing them with data they saw during training.

We compare our method to an adapted version of the highly performing SMILES-based BERT Transformer method BECPred [7]. We re-trained BECPred following the original paper, but modified the fine-tuning input with only compound SMILES instead of reaction SMILES to match our task. As discussed in Section 2.1, comparison to other methods was largely not possible as they use input features that are reliant on having the whole reaction as an input, something we do not have.

Our method is implemented in Python 3.12 and uses a custom version of HiClass 4.13 [39] combined with XGBoost [34] for the hierarchical classification and PyTorch 2.7 [40] for the product prediction. We use the Adam optimiser with a learning rate of $5\times 10^{-4}$ for training the product prediction model. We also use early stopping with a patience of five and restore the weights to the best validation set accuracy when the early stopping triggers.

All the enviPath data is available on enviPath, https://envipath.org/. Our code is available here and the ECMap dataset is available here.

## 4 Results & discussion

In this section, we give the results of our method evaluation. First, Section 4.1 discusses the tuning results from our tuning methodology. Section 4.2 evaluates ECLIPSE with our metrics. Then, Section 4.3 combines the EC number prediction with product prediction and evaluates using the product prediction metrics. Lastly, Section 4.4 gives the results from the case study with the enviPath datasets. Section 3 in the Supplementary material gives an analyses of the runtime performance and efficiency of the different methods.

### 4.1 Tuning results

In Table 4 we present the best model’s parameter configuration for each cross-validation fold. The configurations for both models largely choose the top number of estimators at 500, especially with the H-ECLIPSE in Table 4, where only one fold chooses 200 instead. The maximum depth is more varied, with three and four different values achieving the best performance, in the respective methods. Across both methods, the tolerance is always at zero, meaning only one class will be predicted for any given input. The tuning process has determined there is no benefit to predicting multiple classes as most additional labels would be incorrect leading to a reduction in precision without a corresponding increase in recall, thus, the F1-score drops. This is unsurprising due to the low number of multi-label classes in the ECMap dataset. For the F-ECLIPSE, the number of chains also varied with 10, 20 and 40 values, all performing best on different folds. Overall, we see a bias towards larger, more complex models with high numbers of estimators and high maximum depths.

**Table 4:** ECMap Best Parameters

F-ECLIPSE					H-ECLIPSE		
Est	Depth	Chains	Tol	Fold	Est	Depth	Tol
500	9	10	0.000	0	500	15	0.000
500	12	40	0.000	1	200	12	0.000
500	6	20	0.000	2	500	12	0.000
200	12	40	0.000	3	500	12	0.000
500	15	40	0.000	4	500	15	0.000
500	12	40	0.000	5	500	9	0.000
200	9	10	0.000	6	500	15	0.000
200	12	20	0.000	7	500	12	0.000
500	15	40	0.000	8	500	15	0.000
500	12	10	0.000	9	500	15	0.000

F-ECLIPSE paramters are on the left of the fold number and H-ECLIPSE are on the right. Est is the number of estimators, Depth is the maximum depth and Tol is the tolerance. Chains is the number of chains for F-ECLIPSE

### 4.2 ECLIPSE evaluation

For ECLIPSE model, we give the mean and standard deviation of the precision, recall and F1-score at each EC level and the overall hierarchical version. In Table 5, we see that our proposed methods, F-ECLIPSE and H-ECLIPSE, outperform the baseline BECPred and that H-ECLIPSE has the highest mean across all metrics.

Comparing F-ECLIPSE and H-ECLIPSE to BECPred, the hierarchical metrics show increases in the means of all three metrics, ranging between 2.4 and 2.8 percentage points. H-ECLIPSE and F-ECLIPSE perform very similarly with precision, recall, and F1, all sitting around 93.0%. Looking at the specific EC level metrics, F-ECLIPSE and H-ECLIPSE once again perform similarly to each other but surpass BECPred across all three metrics and levels. We also see that the metrics decrease at higher EC levels for all methods. BECPred sees a drop of almost 11 percentage points between levels one and three, whereas F-ECLIPSE and H-ECLIPSE only drop by four percentage points.

**Table 5:** Performance of BECPred, F-ECLIPSE and H-ECLIPSE on the ECMap dataset

Level	Metric	BECPred	F-ECLIPSE	H-ECLIPSE
H	F1	90.49% ± 0.45	93.02% ± 0.16	**93.20% **± **0**.**20**
	Precision	90.49% ± 0.45	93.14% ± 0.30	**93.20% **± **0**.**20**
	Recall	90.49% ± 0.45	92.89% ± 0.50	**93.20%** ± **0**.**20**
L1	F1	95.66% ± 0.22	96.27% ± 0.12	**96.50%** ± **0**.**16**
	Precision	96.31% ± 0.22	96.92% ± 0.12	**97.15%** ± **0**.**17**
	Recall	95.02% ± 0.23	95.62% ± 0.13	**95.85%** ± **0**.**16**
L2	F1	89.77% ± 0.42	92.45% ± 0.21	**92.62%** ± **0**.**26**
	Precision	91.26% ± 0.44	93.99% ± 0.16	**94.16%** ± **0**.**22**
	Recall	88.32% ± 0.42	90.96% ± 0.27	**91.12%** ± ** 0.30**
L3	F1	84.87% ± 0.57	89.07% ± 0.28	**89.12%** ± ** 0.31**
	Precision	87.10% ± 0.62	91.40% ± 0.22	**91.46%** ± ** 0.26**
	Recall	82.76% ± 0.56	86.84% ± 0.37	**86.89%** ± ** 0.37**

Results are split into hierarchical metrics and per EC level metrics. The highest mean for each metric is bolded

We group the predictions by their true EC level one to further analyse the methods’ performance. Table 6 shows the hierarchical F1 for each EC. We give the full table with precision and recall in Table 1 of the supplementary material. F-ECLIPSE and H-ECLIPSE can outperform BECPred with increases in mean F1 of up to 4.3 percentage points on ECs one through four. ECs five, six and seven have higher standard deviations across all methods, and thus, there is a large overlap between the standard deviations of the results. This is likely due to the small amount of data available for those classes. However, F-ECLIPSE and H-ECLIPSE still achieve higher mean F1 in these ECs with mean F1 increases of 3.9, 0.8 and 5.3 percentage points over BECPred. Between F-ECLIPSE and H-ECLIPSE, the models perform very similarly, with often less than one percentage point difference between them. We see the largest gap between F-ECLIPSE and H-ECLIPSE on EC five, where H-ECLIPSE has a mean F1-score 1.7 percentage points higher. The performance on different ECs generally follows the proportion of each class present, with EC two containing the most data performing the best and EC seven containing the least data performing the worst (Fig. 1a). EC six is an exception where, despite its small proportion in the dataset, all three methods achieve F1-scores over 90%. Overall, these results suggest that ECLIPSE methods can better learn the EC numbers related to a compound compared to BECPred.

**Table 6:** This table gives the hierarchical F1 grouped by EC level one for BECPred, F-ECLIPSE and H-ECLIPSE, on the ECMap dataset

EC	BECPred	F-ECLIPSE	H-ECLIPSE
1	86.67% ± 0.91	90.25% ± 0.57	**91.02%** ± ** 0.50**
2	96.22% ± 0.35	97.22% ± 0.20	**97.27%** ± ** 0.22**
3	84.59% ± 0.67	**88.61%** ± ** 0.45**	88.47% ± 0.44
4	76.40% ± 1.48	**80.51%** ± **1**.**60**	80.01% ± 1.36
5	62.56% ± 4.02	64.70% ± 2.18	**66.42%** ± ** 3.14**
6	90.91% ± 1.19	**91.70%** ± ** 1.72**	91.50% ± 1.36
7	45.37% ± 8.85	50.13% ± 11.82	**50.70%** ± ** 12.31**

The highest mean for each EC level is bolded

### 4.3 Product prediction evaluation

Here, we compare the incorporation of known enzymes, predicted enzymes, and no enzymes into the standard product prediction transformer. We opt to use the H-ECLIPSE model for the predicted EC as it achieved the highest overall F1-score (Table 5). Table 7 shows that including the true enzyme class (True EC) increases the overall mean Top-1 by 1.2 percentage points compared to no EC number. There are similar increases across EC classes one through five. EC six sees a much smaller increase of less than 0.1 percentage points, and EC seven sees a 1.2 reduction in Top-1, however, the increase swings back in favour of True EC for Top-2 and Top-3.

Pred EC performs similarly to No EC in overall performance and EC’s one through five. EC seven sees a drastic decrease in performance with the mean Top-1 dropping by nine percentage points. The performance of Pred EC on each EC class in product prediction is strongly linked to the H-ECLIPSE’s ability to predict that EC class. EC two sees the best result for Pred EC with an increase of 0.8 percentage points over No EC. This corresponds to where H-ECLIPSE performs the best with an F1-score of 97.27%. Following this pattern, EC seven’s drastic drop in performance of nine percentage points between Pred EC and No EC can be explained by H-ECLIPSE’s F1-score of only 50.70% on EC seven.

**Table 7:** Top-K accuracy comparison across True EC, Pred EC, and No EC models for the ECMap dataset. The highest mean for each level one EC is bolded.

EC	Top-K	True EC	Pred EC	No EC
Overall	Top-1	**59.66%** ± ** 1.81**	58.09% ± 1.69	58.46% ± 2.32
	Top-2	**68.68%** ± ** 1.69**	66.75% ± 1.57	67.85% ± 2.04
	Top-3	**72.05%** ± ** 1.69**	69.93% ± 1.54	71.18% ± 1.96
1	Top-1	**49.32%** ± ** 1.71**	47.48% ± 1.75	47.98% ± 2.15
	Top-2	**59.35%** ± ** 2.14**	56.99% ± 2.00	58.12% ± 2.13
	Top-3	**63.82%** ± ** 2.58**	61.18% ± 2.45	62.48% ± 2.15
2	Top-1	**68.32%** ± ** 1.90**	67.85% ± 1.88	67.08% ± 2.74
	Top-2	**76.01%** ± ** 1.37**	75.45% ± 1.34	75.51% ± 2.08
	Top-3	**78.53%** ± ** 1.20**	77.88% ± 1.18	78.04% ± 1.90
3	Top-1	**57.75%** ± ** 2.17**	54.67% ± 1.81	56.85% ± 1.99
	Top-2	**68.37%** ± ** 2.11**	64.59% ± 1.83	67.82% ± 1.94
	Top-3	**72.07%** ± ** 1.93**	68.09% ± 1.49	71.41% ± 1.74
4	Top-1	**51.20%** ± ** 3.14**	48.80% ± 2.77	49.65% ± 3.33
	Top-2	**59.43%** ± ** 3.35**	56.56% ± 2.99	57.47% ± 3.66
	Top-3	**63.33%** ± ** 3.55**	60.11% ± 3.16	61.31% ± 3.51
5	Top-1	**24.53%** ± ** 3.30**	22.66% ± 2.98	22.98% ± 3.95
	Top-2	**33.23%** ± ** 3.36**	30.70% ± 3.01	31.46% ± 3.85
	Top-3	**36.93%** ± ** 3.39**	34.24% ± 3.24	36.10% ± 3.78
6	Top-1	**57.04%** ± ** 4.23**	55.05% ± 4.09	56.95% ± 4.83
	Top-2	**70.15%** ± ** 3.82**	67.68% ± 4.03	69.84% ± 4.10
	Top-3	74.14% ± 3.59	71.28% ± 4.02	**74.21%** ± ** 3.90**
7	Top-1	82.19% ± 11.96	74.40% ± 11.69	**83.41%** ± ** 13.61**
	Top-2	**87.41%** ± ** 8.20**	77.77% ± 10.01	86.93% ± 10.78
	Top-3	**88.99%** ± ** 6.46**	80.29% ± 8.62	88.52% ± 8.37

**Fig. 3:**
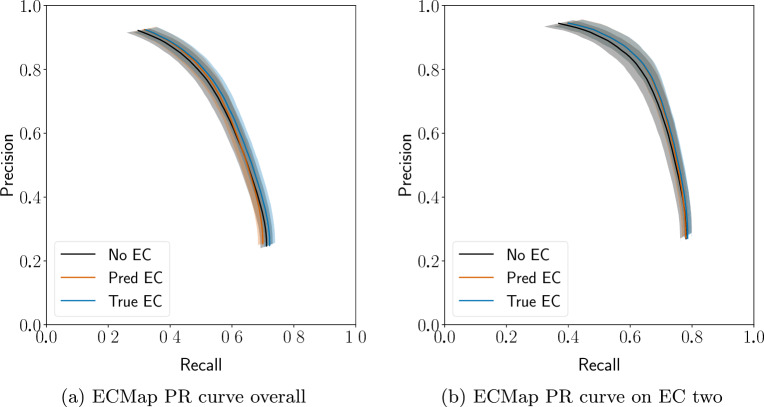
ECMap PR curves overall and on EC class two. Other PR curves by EC level one class are given in the supplementary material

The PR curves in Fig. 3 show the overall product prediction performance and product prediction on EC two. All three methods perform very similarly in the overall plot (Fig. 3a). The True EC and Pred EC achieve a slightly higher peak precision than No EC. True EC also achieves a higher peak recall than No EC. Looking at EC class two (Fig. 3b), we see True EC and Pred EC outperform No EC with higher peak precision and curves consistently more towards the top right. True EC and Pred EC have the most improvement in both figures over No EC in the middle of the PR curve.

In product prediction, the association between data volume and model performance is weaker than in ECLIPSE. Table 7 shows us that higher proportion classes, such as EC one, two and three, perform similarly to lower proportion classes six and seven. Additionally, whereas ECLIPSE performs worse on EC seven, product prediction performs best.

Overall, including EC numbers in reaction prediction can slightly improve prediction performance. However, when using predicted EC numbers, it is clear that errors made by the ECLIPSE can significantly impact the product prediction results.

### 4.4 Case study: enviPath

We use the enviPath dataset as a case study investigating how our method performs on a smaller dataset. We do this in two contexts, retraining with BBD and out-of-the-box inference with Soil, Sludge and BBD. We have to perform pure inference on Soil and Sludge due to their highly limited amount of EC information. However, out-of-the-box inference on BBD allows us to see how our method performs when the test set is less representative of the training set. We split the BBD dataset into train and test sets using the same 10-fold cross-validation method we used with ECMap. We use only BBD for training as it is the sole enviPath package with EC numbers for the reactions. We then retrain BECPred, F-ECLIPSE and H-ECLIPSE.

#### 4.4.1 BBD tuning results

In Table 8 we give the parameters of the best F-ECLIPSE and H-ECLIPSE models on each cross-validation fold. Compared to the parameters for ECMap (Table 4), we see smaller numbers of estimators and smaller maximum depths being chosen. In the H-ECLIPSE parameters shown in Table 8, most estimators are either 20 or 50, and the max depth is mostly six or nine. F-ECLIPSE in Table 8 has generally higher estimator numbers, with a couple at 100 and 200, but has similar maximum depths. For the number of chains in the F-ECLIPSE, this is similar to the ECMap configurations, but it does see three five-chain configurations appear. These lower parameter values lead to less complex models, perhaps avoiding over-fitting on the smaller dataset. Unlike the ECMap dataset, some models choose non-zero values for the tolerance. As shown in Table 1, BBD is a more multi-label dataset than ECMap. Therefore, predicting multiple enzymes leads to an increase in recall and F1-score. However, even in these instances, the chosen tolerance value is small, with the most common value being 0.01, reflecting the fact that BBD is still largely single-label. The choice of non-zero tolerance suggests that with a more multi-labelled dataset, our tuning process would suitably adapt its tolerance to predict multiple EC numbers. Overall, the features of these top models show that our tuning process is indeed adapting to the different datasets.

**Table 8:** BBD best parameters

F-ECLIPSE					H-ECLIPSE		
Est	Depth	Chains	Tol	Fold	Est	Depth	Tol
500	6	20	0.010	0	20	15	0.000
200	6	5	0.010	1	100	9	0.010
200	15	10	0.100	2	20	6	0.025
500	3	5	0.000	3	50	9	0.000
100	6	5	0.000	4	500	9	0.000
20	15	5	0.000	5	50	15	0.000
50	9	10	0.100	6	200	6	0.000
50	15	10	0.000	7	50	15	0.000
50	3	40	0.010	8	50	6	0.025
100	9	20	0.000	9	20	6	0.000

F-ECLIPSE paramters are on the left of the fold number and H-ECLIPSE are on the right. Est is the number of estimators, Depth is the maximum depth and Tol is the tolerance. Chains is the number of chains for F-ECLIPSE

#### 4.4.2 BBD ECLIPSE evaluation

We performed the same evaluation of the models trained on BBD as we did on the ECMap dataset. Table 9 shows that F-ECLIPSE and H-ECLIPSE again outperform BECPred, with H-ECLIPSE performing the best.

H-ECLIPSE and F-ECLIPSE outperform BECPred with a mean F1-score, precision and recall of 7.5 percentage points higher in the hierarchical measures. H-ECLIPSE also achieves a slightly higher mean than F-ECLIPSE, approximately one percentage point. We see results similar to those of the hierarchical level for the three EC levels. H-ECLIPSE achieves means of six, nine and six percentage points higher than BECPred for EC levels one, two and three, respectively. H-ECLIPSE is also consistently the top performing except for recall on level three, where F-ECLIPSE performs slightly better. These results show the same trend as the results on the ECMap dataset, with H-ECLIPSE performing the best and BECPred performing the worst.

**Table 9:** Performance of BECPred, F-ECLIPSE and H-ECLIPSE on the BBD dataset

Level	Metric	BECPred	F-ECLIPSE	H-ECLIPSE
H	F1	53.57% ± 4.03	60.02% ± 3.24	**61.09%** ± ** 3.33**
	Precision	53.57% ± 4.03	59.98% ± 2.92	**61.62%** ± ** 3.60**
	Recall	53.57% ± 4.03	60.08% ± 3.81	**60.59%** ± ** 3.27**
L1	F1	69.70% ± 3.51	74.52% ± 4.13	**75.92%** ± ** 2.80**
	Precision	71.51% ± 3.42	75.46% ± 4.21	**77.78%** ± ** 2.81**
	Recall	68.00% ± 3.73	73.69% ± 4.85	**74.16%** ± ** 3.00**
L2	F1	48.31% ± 3.99	55.87% ± 3.14	**57.45%** ± ** 2.66**
	Precision	50.48% ± 3.98	57.05% ± 4.23	**59.91%** ± ** 3.04**
	Recall	46.33% ± 4.07	55.03% ± 4.42	**55.20%** ± ** 2.56**
L3	F1	44.63% ± 4.87	49.72% ± 3.64	**50.64%** ± ** 3.55**
	Precision	47.22% ± 4.72	51.24% ± 5.18	**53.41%** ± ** 3.67**
	Recall	42.34% ± 5.04	**48.62%** ± ** 4.20**	48.18% ± 3.66

Results are split into hierarchical metrics and per EC level metrics. The highest mean for each metric is bolded

Table 10 breaks down each method’s hierarchical F1-score by EC level one. We give the full table with precision and recall in Table 2 of the supplementary material. EC six and seven are removed as they contain too few instances. We see that F-ECLIPSE achieves the highest mean metrics on most ECs except EC one, where H-ECLIPSE achieves the highest. Grouping by EC greatly reduced the amount of data, leading to very high standard deviations across all methods and ECs. Despite that, we find that F-ECLIPSE and H-ECLIPSE outperform BECPred on every level one EC, with mean F1 scores of up to 27 percentage points higher in EC five. Between F-ECLIPSE and H-ECLIPSE, we see that H-ECLIPSE outperforms on EC by a mean of 3.2 percentage points. On all other ECs, F-ECLIPSE outperforms H-ECLIPSE with a maximum increase of 4.9 percentage points on EC two and a minimum increase of 0.9 percentage points on EC four.

**Table 10:** This table gives the hierarchical F1 grouped by EC level one for BECPred, F-ECLIPSE and H-ECLIPSE, on the BBD dataset

EC	BECPred	F-ECLIPSE	H-ECLIPSE
1	59.72% ± 5.25	63.74% ± 3.27	**66.94%** ± ** 3.96**
2	29.22% ± 19.37	**40.08%** ± ** 20.33**	35.18% ± 21.73
3	52.99% ± 4.29	**57.02%** ± ** 12.24**	55.09% ± 12.04
4	33.71% ± 11.66	**47.30%** ± ** 12.76**	46.41% ± 14.24
5	21.50% ± 7.43	**48.83%** ± ** 21.90**	45.00% ± 19.89

The highest mean for each EC level is bolded

Similar to the ECMap results, classification performance follows the proportion of an EC class in the dataset. We see from Fig. 1b that EC one makes up a substantial portion of the BBD data and that in Table 10 all three methods perform best on it. In contrast, EC two makes up a very small portion of the dataset and sees much lower performance across all methods.

#### 4.4.3 BBD product prediction

We perform production prediction on BBD using the same steps we used with ECMap in Section 4.3. Table 11 gives the overall and EC level one Top-K results. We see that True EC performs the best overall with a 2.3 percentage point gain over No EC. True EC also performs best on EC one, three and four. Looking at Pred EC, it outperforms No EC overall and on ECs three and four. Notably, Pred EC matches True EC’s Top-1 performance on EC four. We see that No EC sometimes outperforms True EC, for example on EC two. However, for both True EC and No EC the product prediction performance is very low indicating that the transformer is has trouble learning this class regardless of EC information. This is likely due to the low amount of EC two data in BBD (see Fig. 1b). More broadly, we again see the lower proportion EC classes perform worse such as EC two and five and higher proportion classes such as EC one and three perform better.

**Table 11:** Top-K accuracy comparison across True EC, Pred EC, and No EC models on the BBD dataset. The highest mean for each level one EC is bolded.

EC	Top-K	True EC	Pred EC	No EC
Overall	Top-1	**20.70% ± 5.21**	18.41% ± 4.14	18.38% ± 4.06
	Top-2	**28.50%** ± ** 6.18**	24.90% ± 5.26	24.01% ± 4.54
	Top-3	**30.77%** ± ** 5.70**	26.66% ± 5.50	27.42% ± 4.22
1	Top-1	**23.53%** ± ** 6.68**	20.35% ± 3.91	22.59% ± 4.96
	Top-2	**31.89%** ± ** 7.81**	27.45% ± 6.25	28.00% ± 5.61
	Top-3	**34.30%** ± ** 7.52**	29.14% ± 6.46	30.98% ± 5.79
2	Top-1	3.67% ± 7.77	3.67% ± 7.77	**4.43%** ± ** 10.04**
	Top-2	5.10% ± 8.31	3.67% ± 7.77	**6.10%** ± ** 10.60**
	Top-3	7.76% ± 10.84	6.33% ± 10.82	**9.19%** ± ** 12.59**
3	Top-1	**20.32%** ± ** 8.19**	19.18% ± 8.94	13.24% ± 6.53
	Top-2	**28.74%** ± ** 9.14**	26.08% ± 8.60	20.81% ± 9.95
	Top-3	**30.32%** ± ** 8.44**	27.67% ± 9.39	25.49% ± 9.73
4	Top-1	**9.77%** ± ** 7.15**	**9.77%** ± ** 7.15**	5.79% ± 6.69
	Top-2	**18.21%** ± ** 11.98**	17.10% ± 10.89	10.88% ± 10.78
	Top-3	**19.75%** ± ** 10.87**	17.87% ± 11.03	13.47% ± 9.46
5	Top-1	2.86% ± 6.02	1.43% ± 4.52	**5.93%** ± ** 9.87**
	Top-2	**12.36%** ± ** 16.35**	8.43% ± 11.27	8.43% ± 11.27
	Top-3	**16.36%** ± ** 17.82**	8.43% ± 11.27	14.36% ± 15.89

**Fig. 4:**
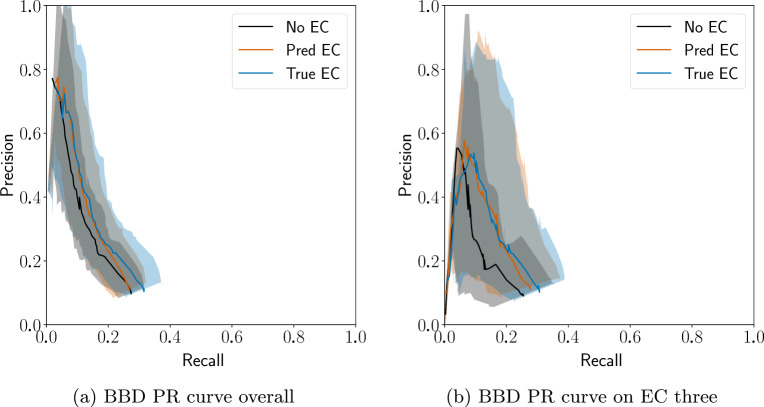
BBD PR curves overall and on EC class three. Other PR curves by EC level one class are given in the supplementary material

The BBD PR curves in Fig. 4 again show True EC and Pred EC outperforming No EC. The overall PR curve in Fig. 4a shows them both having better curves, achieving higher precision than No EC at all recall values. Interestingly, Pred EC maintains the same precision as True EC up to a recall of about 0.2 before its precision drops. Focussing on the EC three PR curve in Fig. 4b.

Overall, these results match those seen in the ECMap results. It is especially the case that the H-ECLIPSE performance greatly impacts product prediction performance with Pred EC’s results on ECs two and five being particularly impacted.

#### 4.4.4 BBD inference

**Fig. 5:**
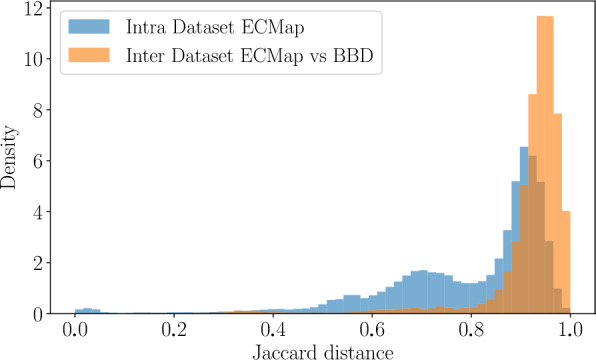
The intra and inter Jaccard distance of ECMap and BBD

To evaluate the generalisation of our method, we use BBD as a test set after training on ECMap. We computed the intra- and inter-dataset distances shown in Fig. 5 to show how dissimilar the two datasets are. The intra-dataset distance is the distance between each point in ECMap and every other point in ECMap. In contrast, the inter-dataset distance is from each point in BBD to each point in ECMap. We see that points within BBD tend to be farther from points in ECMap than points within ECMap are from each other. This suggests that BBD indeed differs from ECMap in distribution and can act as an adequate out-of-distribution performance assessment.

Table 12 shows the prediction performance of H-ECLIPSE compared to F-ECLIPSE and BECPred. As these models were trained on the entire ECMap dataset and tested on BBD, they are single runs, and therefore, no mean is available. Overall, we see a significant drop compared to the BBD cross-validation results in Table 9. However, H-ECLIPSE still outperforms both BECPred and F-ECLIPSE, with hierarchical F1-scores 7.1 and 6.6 percentage points higher, respectively. This drop in performance is expected, given the difference in data distribution.

**Table 12:** Performance of BECPred, F-ECLIPSE and H-ECLIPSE trained on ECMap using BBD as a test set

Level	Metric	BECPred	F-ECLIPSE	H-ECLIPSE
H	F1	12.73%	13.28%	**19.89%**
	Precision	13.33%	13.64%	**20.83%**
	Recall	12.18%	12.94%	**19.04%**
L1	F1	18.47%	21.43%	**33.73%**
	Precision	19.17%	21.95%	**35.00%**
	Recall	17.83%	20.93%	**32.56%**
L2	F1	11.93%	13.08%	**18.18%**
	Precision	14.94%	16.87%	**21.00%**
	Recall	9.92%	10.69%	**16.03%**
L3	F1	**11.32%**	9.71%	10.53%
	Precision	**15.38%**	13.89%	12.77%
	Recall	**8.96%**	7.46%	**8.96%**

Results are split into hierarchical metrics and per EC level metrics. The highest for each metric is bolded

#### 4.4.5 Soil and sludge inference

For performing inference, we first tune our H-ECLIPSE with the ECMap data following the tuning process in Section 3.3.4. Once the best parameters have been found, we retrain that configuration with all ECMap data, including the validation set usually left out in the tuning process.

**Fig. 6:**
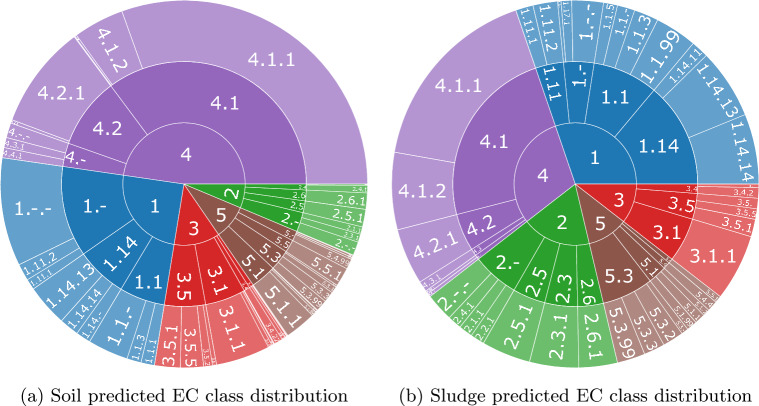
Distribution of predicted EC numbers in Soil (**a**) and Sludge (**b**). We show the top three most common classes at the second and third level and group the rest into the dash. The colour segments each of the seven level one EC numbers

Figure 6 shows the predicted EC classes for Soil (Fig. 6a) and Sludge (6b). We see similar results for both datasets, with level one EC classes one and four being the majority of predictions. However, some differences exist, Soil sees EC two as the minority class, whereas EC three is the minority for Sludge. Interestingly, the model predicted no data in ECs six or seven. Soil and Sludge would likely have characteristics similar to BBD, which contains very few EC six and seven. Therefore, it seems reasonable that Soil and Sludge would have little or no compounds that interact with those EC classes.

**Fig. 7:**
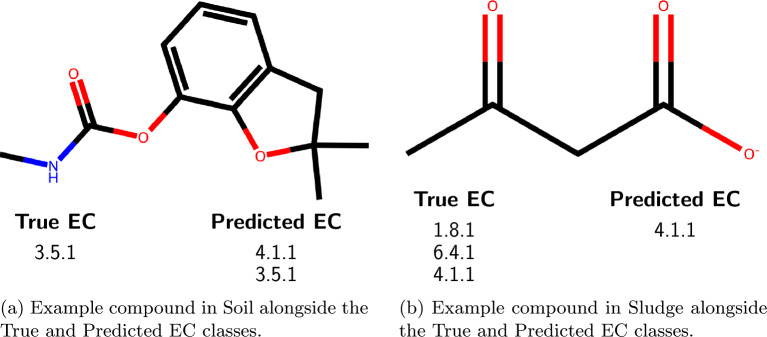
Example compounds from Soil (**a**) and Sludge (**b**) with the true and predicted EC numbers

In Fig. 7 we show one example compound from Soil and Sludge with the true and predicted EC numbers. In the Soil example (Fig. 7a) the ECLIPSE predicts two EC numbers 4.1.1 and 3.5.1 and the true EC is 3.5.1. For Sludge (Fig. 7b) the ECLIPSE predicts only one EC number, 4.1.1 whereas the true set contains three EC numbers, 1.8.1, 6.4.1 and 4.1.1. In both these examples the predicted EC number is present in the true EC list.

After predicting EC numbers for Soil, we performed product prediction. We compare training our product prediction method with the predicted EC numbers and no EC numbers. Here, we have to train the product prediction model with the predicted EC numbers as no true EC numbers are available. We only do this on Soil, as Sludge contains too few reactions. In Table 13, we see Pred EC outperforms No EC overall and all level one ECs by 1.5 percentage points in Top-1.

**Table 13:** Top-K accuracy comparison between Pred EC, and No EC models on the soil dataset. The highest mean for each level one EC is bolded.

EC	Top-K	Pred EC	No EC
Overall	Top-1	**34.30%** ± ** 2.67**	32.79% ± 3.24
	Top-2	**46.65%** ± ** 5.39**	43.23% ± 4.52
	Top-3	**51.52%** ± ** 5.88**	48.62% ± 5.15
1	Top-1	**35.09%** ± ** 4.07**	33.47% ± 5.41
	Top-2	**45.72%** ± ** 5.81**	44.80% ± 3.93
	Top-3	**51.37%** ± ** 5.88**	50.10% ± 3.23
2	Top-1	**25.38%** ± ** 9.61**	23.78% ± 9.06
	Top-2	**39.81%** ± ** 14.24**	38.97% ± 13.75
	Top-3	**45.32%** ± ** 13.49**	42.88% ± 12.70
3	Top-1	**27.01%** ± ** 8.50**	25.31% ± 10.56
	Top-2	**41.91%** ± ** 15.59**	36.08% ± 15.41
	Top-3	**48.24%** ± ** 16.74**	41.10% ± 17.23
4	Top-1	**38.65%** ± ** 5.16**	37.42% ± 6.24
	Top-2	**51.92%** ± ** 6.17**	45.34% ± 6.65
	Top-3	**55.47%** ± ** 6.89**	52.23% ± 7.36
5	Top-1	**35.05%** ± ** 13.48**	33.50% ± 12.77
	Top-2	**44.94%** ± ** 13.70**	44.80% ± 12.30
	Top-3	**48.28%** ± ** 12.93**	46.54% ± 14.48

**Fig. 8:**
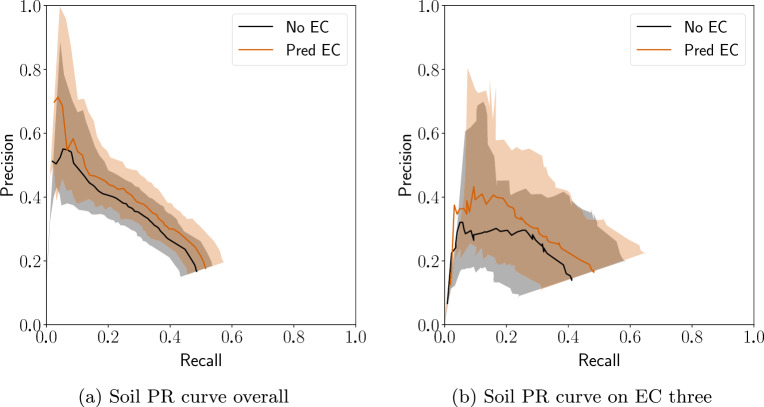
Soil PR curves overall and on EC class three. Other PR curves by EC level one class are given in the n material

The PR curves in Fig. 8 report similar results. Figure 8a shows that Pred EC outperforms No EC across the entire curve, with a higher peak precision of 0.7 compared to 0.55 and a higher peak recall of 0.55 compared to 0.45. Specifically looking at EC three (Fig. 8b), Pred EC again achieves a higher precision of 0.4 compared to 0.3 and a higher recall of 0.5 compared to 0.4.

In the ECMap and BBD results, we noted that Pred EC performed best when the H-ECLIPSE performed best and could only outperform No EC in its best-performing ECs. Subsequently, seeing Pred EC consistently outperform No EC suggest that the EC classes predicted by the H-ECLIPSE for Soil are reasonable.

### 4.5 Results summary

Summarising our results, we draw the following conclusions. Across the ECMap and BBD datasets, our H-ECLIPSE outperforms both F-ECLIPSE and BECPred. This shows that structuring EC number classification as a hierarchical classification problem is better than performing a ‘flat’ classification. Additionally, the performance over BECPred shows that tree-based methods like XGBoost can outperform the popular BERT-style methodologies.

Our product prediction results show that including enzyme information in the transformer model input can improve product prediction performance. This is especially the case on smaller datasets, as we see BBD and Soil gain the most from this additional information. These datasets are harder for the product prediction model to learn due to their limited size compared to larger ones, such as ECMap. Therefore, any relevant extra information, such as EC numbers, can be beneficial. We also note that when using predicted EC numbers, it is important to be aware of where the EC classification model performs poorly, as we find that when the predicted EC is wrong, the product prediction results are detrimentally impacted.

## 5 Limitations & future work

We only tune a small subset of hyperparameters, maximum depth, number of estimators, tolerance and number of chains (for F-ECLIPSE). This limitation is due to computational limitations preventing us from running the tens of thousands of combinations created by including other parameters. Future work could expand tuning to include more parameters and use a more intelligent tuning process that only focuses on the most promising hyperparameter combinations. Bayesian optimisation or genetic algorithms could be candidates.

Following the hyperparameters, we only used the Morgan fingerprint with the default values provided by RDKit. Future work could investigate whether other fingerprints, such as MACCS, or deep learning embeddings from methods such as ChemBerta [41], provide better information for the classifiers.

Concatenating EC numbers to the SMILES is a simplistic way to incorporate enzyme knowledge and may create some interference for the Transformer. Creating deep learning embeddings of the enzyme information to add to SMILES embeddings could be a better way of incorporating this information. Alternatively, using a mixture of experts’ style method, where different product prediction models predict reactions caused by different ECs, could also improve overall product prediction performance. Additionally, we observed that including predicted EC numbers does not always improve product prediction, especially in EC classes where ECLIPSE predicts poorly. Future work combining predicted EC numbers with product prediction could examine mechanisms to mitigate error propagation such as making the EC prediction uncertainty-aware.

## 6 Conclusion

We proposed ECLIPSE, a framework for training and evaluating a hierarchical multi-label classifier to predict associations between enzyme commission numbers and chemical compounds. Experimental evaluations on the ECMap and BBD datasets show that formulating enzyme commission number classification as a hierarchical problem yields better results than a ‘flat’ classification. ECLIPSE outperforms the existing enzyme prediction method, BECPred, with a mean hierarchical F1-score of up to 93.2% on the ECMap dataset. We also alleviate the reliance on product information to predict enzyme commission numbers for chemical reactions. Moreover, incorporating enzyme information is beneficial for product prediction, especially in smaller datasets such as enviPath’s BBD and Soil, where we observe the most significant gains. This research supports ongoing efforts to reduce the environmental impact of chemicals by improving the knowledge and performance of chemical reaction-prediction methods.

## Supplementary Information

Supplementary file 1.

## Data Availability

The data for reproducing the results is available at https://doi.org/10.5281/zenodo.17489235 and the code is available at https://github.com/MyCreativityOutlet/chem-eclipse

## Abbreviations

EC: Enzyme commission
SMILES: Simplified molecular input line entry system
ECLIPSE: Enzyme class learning and interaction prediction systEm
LCL: Local classifier per level
LCPN: Local classifier per parent node
LCN: Local classifier per node
BBD: Biocatalysis/biodegradation database
H-ECLIPSE: Hierarchical ECLIPSE
F-ECLIPSE: Flat ECLIPSE
ECC: Ensemble of classifier chains
PR: Precision-recall
