# Supplementary material for: Predicting enzyme-compound associations for enzyme-catalysed reactions
Source: J Cheminform. 2026 Apr 22;18:77. doi: 10.1186/s13321-026-01190-w (PMC13267362; doi:10.1186/s13321-026-01190-w)
Supplement: Supplementary file 1 [file 13321_2026_1190_MOESM1_ESM.pdf]

Supplementary: Predicting Enzyme-Compound Associations for Enzyme-Catalysed Reactions

Liam Brydon-Brown[1,2*], Gillian Dobbie[1], Katerina Taškova[1], Jörg Simon Wicker[1,2]

[1]School of Computer Science, University of Auckland, Auckland, New Zealand.

[2]enviPath Limited, Auckland, New Zealand.

*Corresponding author(s). E-mail(s): lbry121@aucklanduni.ac.nz;

1 ECLIPSE Performance By Level One EC

Here we give the full tables of the ECLIPSE performance. ECMap is shown in Table 1 and BBD is shown in Table 2.

2 Product Prediction By Level One EC

Here we give the precision-recall (PR) curves for product prediction grouped by level one EC across all datasets. Figure 1 shows the ECMap plots. Figure 2 shows the BBD plots. Figure 3 shows the Soil plots.

3 Runtime

We benchmarked the inference runtime performance of all three methods across batch sizes 1, 10, 100, and 1000. In Figure 4 we see that H-ECLIPSE has a runtime over 16 times quicker than BECPred and F-ECLIPSE for batch sizes 1, 10 and 100. At a batch size of 1000, this drops to four times quicker. Due to its hierarchical structure, H-ECLIPSE only needs to use the positively predicted branch(es) classifiers rather than all its classifiers like F-ECLIPSE. Additionally, the ECC comprising F-ECLIPSE contains significantly more classifiers than H-ECLIPSE. Both these factors lead to

EC	Metric	BECPred	F-ECLIPSE	H-ECLIPSE
1	F1	86.67% ± 0.91	90.25% ± 0.57	**91.02% ± 0.50**
	Precision	86.67% ± 0.91	90.56% ± 0.60	**91.02% ± 0.50**
	Recall	86.67% ± 0.91	89.97% ± 1.34	**91.02% ± 0.50**
2	F1	96.22% ± 0.35	97.22% ± 0.20	**97.27% ± 0.22**
	Precision	96.22% ± 0.35	97.22% ± 0.20	**97.27% ± 0.22**
	Recall	96.22% ± 0.35	97.22% ± 0.20	**97.27% ± 0.22**
3	F1	84.59% ± 0.67	**88.61% ± 0.45**	88.47% ± 0.44
	Precision	84.59% ± 0.67	**88.61% ± 0.45**	88.47% ± 0.44
	Recall	84.59% ± 0.67	**88.61% ± 0.45**	88.47% ± 0.44
4	F1	76.40% ± 1.48	**80.51% ± 1.60**	80.01% ± 1.36
	Precision	76.40% ± 1.48	**80.51% ± 1.60**	80.01% ± 1.36
	Recall	76.40% ± 1.48	**80.51% ± 1.60**	80.01% ± 1.36
5	F1	62.56% ± 4.02	64.70% ± 2.18	**66.42% ± 3.14**
	Precision	62.56% ± 4.02	64.70% ± 2.18	**66.42% ± 3.14**
	Recall	62.56% ± 4.02	64.70% ± 2.18	**66.42% ± 3.14**
6	F1	90.91% ± 1.19	**91.70% ± 1.72**	91.50% ± 1.36
	Precision	90.91% ± 1.19	**91.70% ± 1.72**	91.50% ± 1.36
	Recall	90.91% ± 1.19	**91.70% ± 1.72**	91.50% ± 1.36
7	F1	45.37% ± 8.85	50.13% ± 11.82	**50.70% ± 12.31**
	Precision	45.37% ± 8.85	50.13% ± 11.82	**50.70% ± 12.31**
	Recall	45.37% ± 8.85	50.13% ± 11.82	**50.70% ± 12.31**

Table 1: Performance of BECPred, F-ECLIPSE and H-ECLIPSE on the ECMap dataset. The hierarchical F1, precision and recall are given, grouped by EC level one. The highest mean for each metric is bolded.

H-ECLIPSE's highly efficient inference runtime. All three methods handle increasing batch sizes very well with minimal increase in inference runtimes. This suggests that these methods are highly parallelised. BECPred and F-ECLIPSE both see almost zero increase in runtime as the batch size increases.

EC	Metric	BECPred	F-ECLIPSE	H-ECLIPSE
	F1	59.72% ± 5.25	63.74% ± 3.27	**66.94% ± 3.96**
1	Precision	59.72% ± 5.25	64.53% ± 2.70	**67.71% ± 4.52**
	Recall	59.72% ± 5.25	63.03% ± 4.08	**66.26% ± 3.97**
	F1	29.22% ± 19.37	**40.08% ± 20.33**	35.18% ± 21.73
2	Precision	29.22% ± 19.37	**40.86% ± 20.99**	35.74% ± 22.11
	Recall	29.22% ± 19.37	**39.48% ± 19.99**	34.73% ± 21.52
	F1	52.99% ± 4.29	**57.02% ± 12.24**	55.09% ± 12.04
3	Precision	52.99% ± 4.29	**57.03% ± 12.70**	55.70% ± 11.80
	Recall	52.99% ± 4.29	**57.10% ± 11.94**	54.56% ± 12.31
	F1	33.71% ± 11.66	**47.30% ± 12.76**	46.41% ± 14.24
4	Precision	33.71% ± 11.66	**47.30% ± 12.76**	46.41% ± 14.24
	Recall	33.71% ± 11.66	**47.30% ± 12.76**	46.41% ± 14.24
	F1	21.50% ± 7.43	**48.83% ± 21.90**	45.00% ± 19.89
5	Precision	21.50% ± 7.43	**48.83% ± 21.90**	45.00% ± 19.89
	Recall	21.50% ± 7.43	**48.83% ± 21.90**	45.00% ± 19.89

Table 2: Performance of BECPred, F-ECLIPSE and H-ECLIPSE on the BBD dataset. The hierarchical F1, precision and recall are given, grouped by EC level one. The configuration of Algorithm 1 removed EC classes six and seven as they were too small. The highest mean for each metric is bolded.

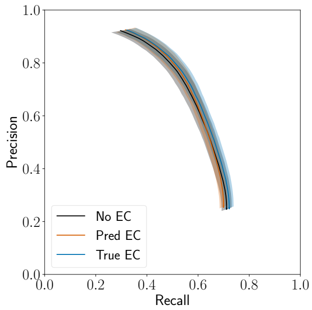

(a) ECMap PR curve overall

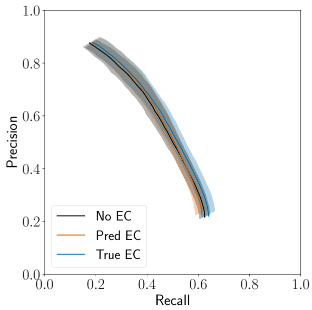

(b) ECMap PR curve on EC one

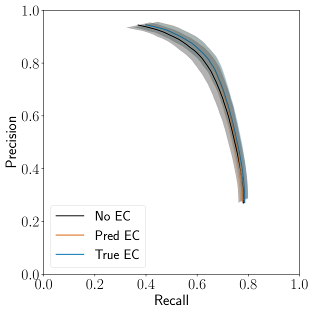

(c) ECMap PR curve on EC two

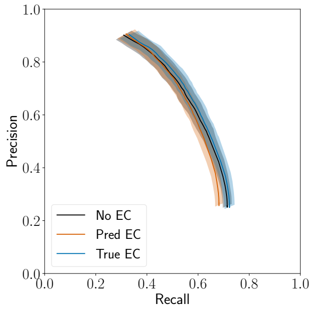

(d) ECMap PR curve on EC three

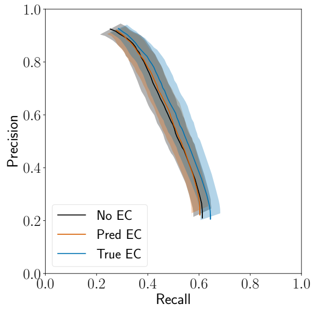

(e) ECMap PR curve on EC four

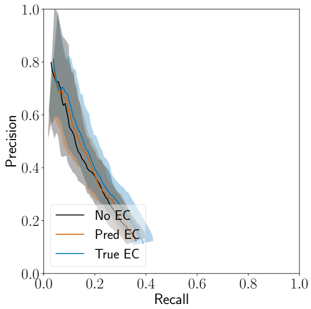

(f) ECMap PR curve on EC five

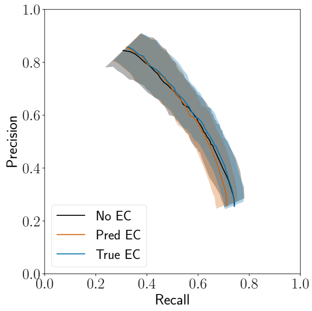

(g) ECMap PR curve on EC six

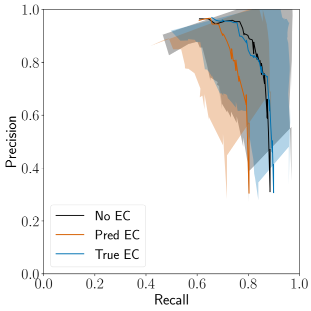

(h) ECMap PR curve on EC seven

Fig. 1: ECMap PR curves overall and by EC level one class

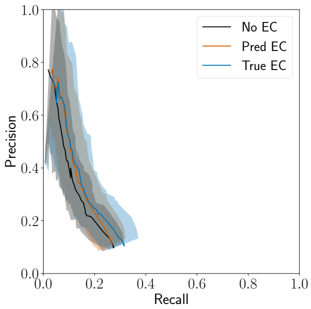

(a) BBD PR curve overall

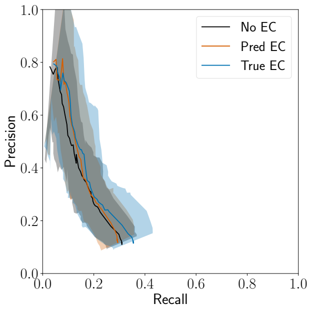

(b) BBD PR curve on EC one

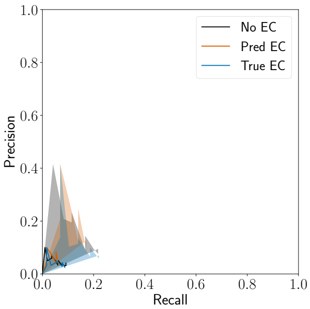

(c) BBD PR curve on EC two

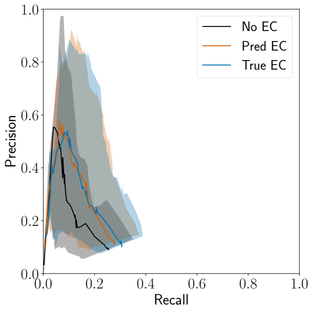

(d) BBD PR curve on EC three

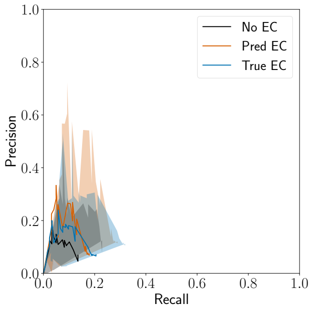

(e) BBD PR curve on EC four

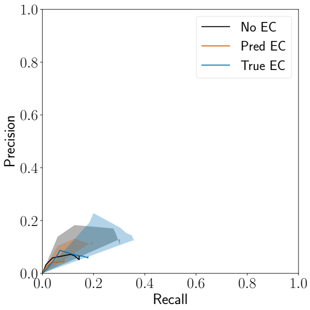

(f) BBD PR curve on EC five

Fig. 2: BBD PR curves overall and by EC level one class

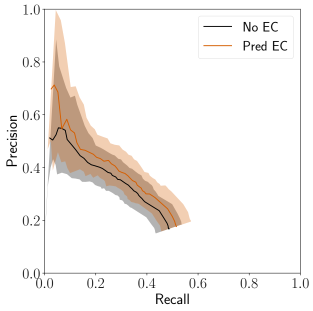

(a) Soil PR curve overall

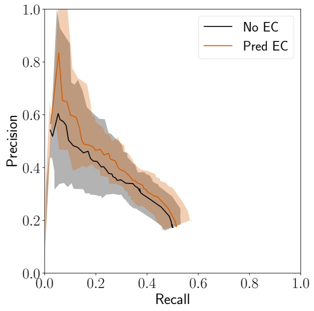

(b) Soil PR curve on EC one

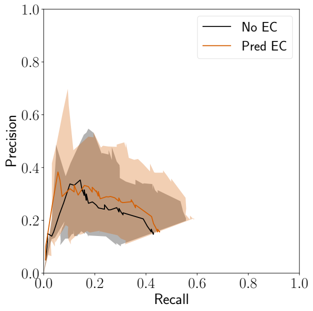

(c) Soil PR curve on EC two

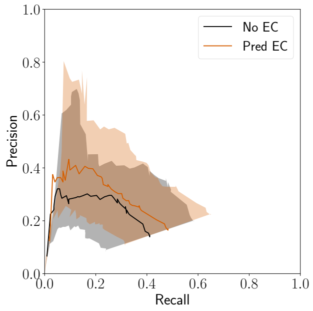

(d) Soil PR curve on EC three

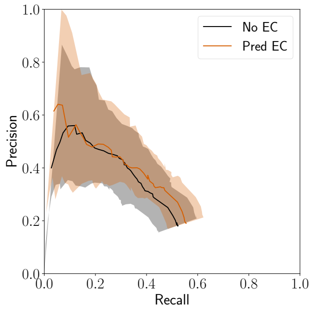

(e) Soil PR curve on EC four

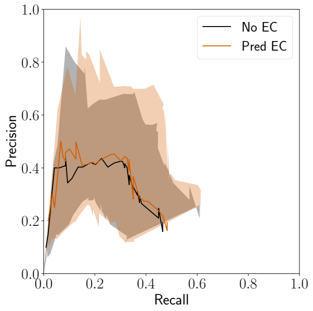

(f) Soil PR curve on EC five

Fig. 3: Soil PR curves overall and by EC level one class

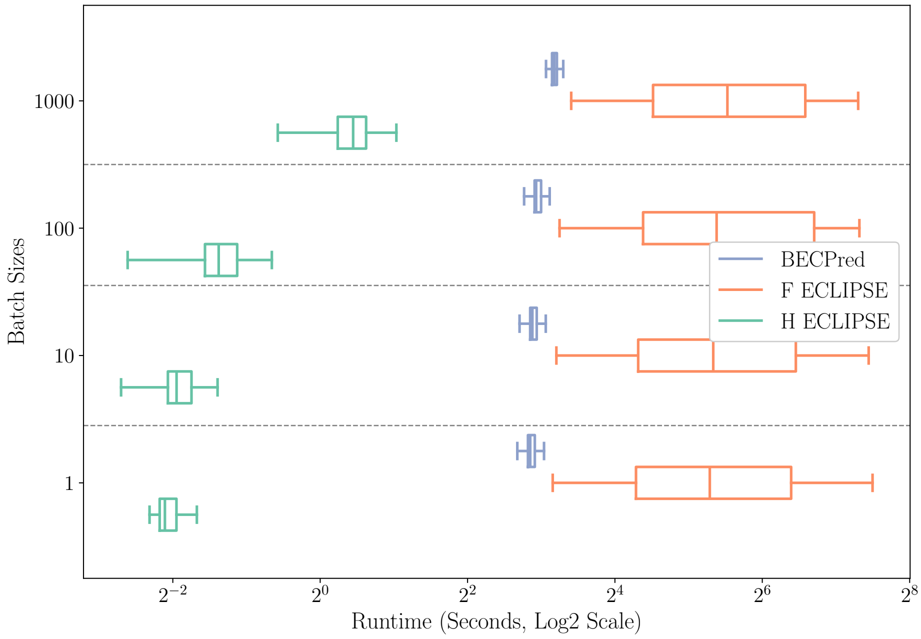

Fig. 4: Inference runtimes of BECPred, F-ECLIPSE and H-ECLIPSE on different batch sizes. Note the Log2 scale of the X-axis.
